# Antinociceptive Effect of the Essential Oil Obtained from the Leaves of *Croton cordiifolius* Baill. (Euphorbiaceae) in Mice

**DOI:** 10.1155/2015/620865

**Published:** 2015-03-02

**Authors:** Lenise de Morais Nogueira, Monalisa Ribeiro da Silva, Simone Maria dos Santos, Julianna Ferreira Cavalcanti de Albuquerque, Igor Cavalcanti Ferraz, Thaíse Torres de Albuquerque, Carlos Renato França de Carvalho Mota, Renata Mendonça Araújo, Glauce Socorro de Barros Viana, René Duarte Martins, Alexandre Havt, Rafael Matos Ximenes

**Affiliations:** ^1^Departamento de Fisiologia e Farmacologia, Universidade Federal do Ceará, Rua Coronel Nunes de Melo 1127, 60430-270 Fortaleza, CE, Brazil; ^2^Departamento de Antibióticos, Universidade Federal de Pernambuco, Rua Professor Artur de Sá, s/n, 50740-520 Recife, PE, Brazil; ^3^Centro Acadêmico de Vitória, Universidade Federal de Pernambuco, Rua Alto do Reservatório, s/n, 55608-680 Vitória de Santo Antão, PE, Brazil; ^4^Instituto de Química, Universidade Federal do Rio Grande do Norte, Avenida Senador Salgado Filho 3000, 50078-970 Natal, RN, Brazil

## Abstract

*Croton cordiifolius* Baill. is a shrub known as “quebra-faca” and is used to treat inflammation, pain, wounds, and gastrointestinal disturbances in the semiarid region in the northeast of Brazil. In an ethnobotanical survey in the state of Pernambuco, “quebra-faca” use was cited in 33% of the interviews. Thus, we decided to evaluate the antinociceptive effects of the essential oil from *C. cordiifolius* (CcEO). Chemical analysis by gas chromatography-mass spectrometry revealed 1,8-cineole (25.09%) and *α*-phellandrene (15.43%) as major constituents. Antinociceptive activity was evaluated using murine models of chemically induced pain (writhing induced by acetic acid, formalin, capsaicin, and glutamate tests). Opioid and central nervous systems (CNS) involvement were also investigated. Regarding antinociceptive activity, CcEO (50 and 100 mg/kg) reduced the number of writhing responses induced by acetic acid and decreased the licking times in both phases of the formalin test. CcEO also was evaluated in capsaicin- and glutamate-induced nociception. While no effect was observed in the capsaicin test, CcEO (100 mg/kg) was effective in the glutamate test. Naloxone, an opioid antagonist, did not affect the antinociceptive activity of CcEO in writhing test. In conclusion, the antinociceptive effect of CcEO could be explained, at least in part, by inhibition of the glutamatergic system.

## 1. Introduction

*Croton* is the second largest genus of the Euphorbiaceae family with approximately 1,200 species.* Croton* has a pantropical distribution, mainly in the Americas, and about 350 species are found in Brazil [1].* Croton* spp. are rich sources of secondary metabolites including alkaloids, flavonoids, and terpenoids, which are responsible for the therapeutic properties of many* Croton* species [2].

Many pharmacological activities are related to* Croton* spp., such as antinociceptive, anti-inflammatory, wound healing, anticancer, and antimicrobial activities [3–8]. However, the pharmacological potential of many species remains completely unexplored.

Three* Croton* species are known as “quebra-faca” in the semiarid region of the northeast of Brazil:* Croton cordiifolius* Baill.,* Croton conduplicatus* Kunth., and* Croton heliotropiifolius* Kunth. [9]. Beside sharing the same popular name, these species are used to treat the same medical conditions, such as general inflammation, pain, and gastrointestinal disturbances [10, 11].

To date, no data about the chemistry and pharmacological properties of* Croton cordiifolius* Baill. could be found in literature. Thus, we performed an ethnobotanical survey, and after analyzing the data, we decided to investigate the role of the essential oil obtained from the leaves of* C. cordiifolius* (CcEO) in its claimed antinociceptive activity, since essential oils, in general, exhibit antimicrobial, anti-inflammatory, and antinociceptive properties [7].

## 2. Material and Methods

### 2.1. Ethnobotanical Survey

Ethnobotanical data were collected through semistructured interviews in two phases [12]. Informed free consent terms were obtained from those who offered to participate in the study, following the legal and ethical regulations set out in the 196/96 resolution from the Ethics Committee on Research of the Ministry of Health, Brazil. In the first phase, the following guiding question was used: what medicinal plants to treat inflammation and pain do you know about? Subsequently, details concerning the part of the plant used, preparation method, indications, and contraindications of each species mentioned were recorded. In the second phase, after analysis of data from the first phase, one specimen of* C. cordiifolius* was shown to the interviewees for recognition. Data were collected between January and March of 2011 in the urban and rural areas of the cities of Salgueiro, Terra-Nova, Parnamirim, and Serrita, which are all located in the state of Pernambuco, in the central region of the northeast of Brazil. One hundred people of both sexes were interviewed.

The use value (UV) of* C. cordiifolius* was calculated according to the formula described by Rossato et al. [13]:

(1) $$\begin{array}{c} \text{UV}=\frac{\Sigma Ui}{n}, \end{array}$$

where *Ui* = the number of uses cited by each informant and *n* = the total number of informants.

### 2.2. Plant Material

Leaves of* Croton cordiifolius* Baill. were collected in the morning during the flowering period (April 2011) in the rural area of Salgueiro, Pernambuco, Brazil (−8° 04′ 27′′ S, −39° 07′ 09′′ W, 420 m). The botanical material was authenticated by botanist Maria Olívia de Oliveira Cano of the Herbarium of the Agronomic Institute of Pernambuco (IPA). A voucher specimen was deposited under the number 85,609.

### 2.3. Essential Oil Extraction

Fresh leaves were submitted immediately for hydrodistillation at 96°C for 2 h in a Clevenger-type apparatus. The essential oil subsequently was dried over anhydrous sodium sulfate while protected from light and frozen at −20°C until use.

For the pharmacological assays, the density of the essential oil was determined using a 1 mL pycnometer, and then the doses were calculated. CcEO was suspended in 0.5% Cremophor (Sigma-Aldrich, St. Loius, MO, USA) and sonicated before use.

### 2.4. Chemical Analysis

Gas chromatography and mass spectrometry (GC/MS) were conducted at the Technological Development Park of the Federal University using a GC/MS QP 5050A (Shimadzu, Kyoto, Japan) and an Agilent DB-5ms nonpolar capillary column (50 m × 0.25 mm × 0.25 *μ*m). The oven temperature was programmed at 70°C with an increase of 4°C/min until 280°C was reached and then maintained for 15 min. The carrier gas was helium, with a constant flow rate of 1.4 mL/min. The temperature of the ionization source was maintained at 280°C, ionization energy at 70 eV, and ionization current at 0.7 kV. Mass spectra were recorded from 30 to 450 m/z. Individual components were identified by matching their 70 eV mass spectra with those of the spectrometer database by using the Wiley L-Built library and by comparing their retention indices and fragmentation patterns with those of the NIST [14] MS library and those reported in the literature [15], respectively. The retention indices were compared with those obtained by Craveiro et al. [16] for other Euphorbiaceae species and simulated using the method described by Alencar et al. [17]. ^1^H and ^13^C NMR were recorded on a Bruker Avance DRX-300 (300 MHz for ^1^H and 75 MHz for ^13^C); chemical shifts are given in ppm relative to residual CHCl_3_ (7.27) and to the central peak of the triplet related to CDCl_3_ carbon (77.2 ppm).

### 2.5. Chemicals and Drugs

Acetic acid and 37% formaldehyde were from Merck (Brazil, São Paulo). Capsaicin, glutamate, indomethacin, and polyethoxylated castor oil (Cremophor) were from Sigma (USA, St. Louis). Morphine and naloxone were purchased from Cristália (Brazil, São Paulo), while diazepam was from União Química (Brazil, São Paulo). All other reagents and substances were of analytical grade.

### 2.6. Animals

Male Swiss mice (25–30 g, *n* = 8) were provided by the Animal Facility of the Federal University of Pernambuco. The animals were housed and kept in a room with controlled temperature (23 ± 2°C) under a 12/12 h light/dark cycle with food and water* ad libitum*. Experiments were carried out according to the Guide for the Care and Use of Laboratory Animals of the US Department of Health and Human Services (NIH publication number 85-23, revised in 1985). The project had been previously approved by the Animal's Ethics Committee of Federal University of Pernambuco (number 23076.020508/2010-26).

### 2.7. Acute Toxicity

A limit test of 1,000 mg/kg was performed according to OECD 425 guideline [18] to determine the acute toxicity of the essential oil using few animals. The CcEO (1,000 mg/kg, i.p.) was administered to one animal followed by 24 h of observation. As this animal did not die, two more animals were administered with CcEO (1,000 mg/kg, i.p.) under the same conditions. A total of five animals were used. After the long-term observation of 14 days, the animals were killed and gross necropsy carried out.

### 2.8. Acetic Acid Induced Writhing

The acetic acid induced writhing was performed according to the protocol described by Koster et al. [19] with some modifications [8].* C. cordiifolius* essential oil (50 and 100 mg/kg, i.p.) or indomethacin (10 mg/kg, i.p.) was given 30 min before the administration of a 0.6% acetic acid solution (10 mL/kg, i.p.). After 10 min, the number of writhing was counted for 20 min by a blind observer.

### 2.9. Formalin Induced Nociception

Twenty microliters of 1% formalin solution were administered (s.c.) to each mouse's right hind paw. Licking time was recorded from 0 to 5 min (phase 1, neurogenic) and from 20 to 25 min (phase 2, inflammatory) after formalin injection [20]. The animals were treated with vehicle (10 mL/kg, i.p.), morphine (7.5 mg/kg, i.p.), or CcEO (50 and 100 mg/kg, i.p.) 30 min before the formalin administration.

### 2.10. Capsaicin Induced Nociception

After an acclimatization period of 20 min, the animals received 20 *μ*L of capsaicin (1.6 *μ*g/paw freshly prepared in PBS) by intraplantar injection in the right hindpaw. Animals were observed individually for 5 min following capsaicin injection. The amount of time spent licking the injected paw was timed and considered as indicative of nociception [21]. The animals were treated with vehicle (10 mL/kg, i.p.) or CcEO (50 and 100 mg/kg, i.p.) 30 min before the capsaicin administration.

### 2.11. Glutamate Induced Nociception

Animals received an intraplantar injection of 20 *μ*L of glutamate (20 *μ*mol/paw prepared in PBS) in the right hindpaw. The amount of time they spent licking the injected paw during the observation period of 15 min following glutamate injection was recorded with a chronometer and considered indicative of nociception [22]. Animals were treated with vehicle (10 mL/kg, i.p.) or CcEO (50 and 100 mg/kg, i.p.) 30 min before the glutamate injection.

### 2.12. Evaluation of Opioid Involvement

To assess the possible involvement of the opioid system in the antinociceptive effect of the essential oil, mice were treated with naloxone (1 mg/kg, i.p.) 15 minutes prior the administration of the CcEO (100 mg/kg, i.p.), morphine (7.5 mg/kg, i.p.), or vehicle (10 mL/kg, i.p.). Thirty minutes later, the animals received 0.6% acetic acid solution (10 mL/kg, i.p.). After 10 min, the number of writhing was counted during 20 min [23].

### 2.13. Evaluation of the Locomotor Activity

The open-field test was used to evaluate the effect of* C. cordiifolius* essential oil on locomotor activity of mice. An acrylic box (transparent walls and black floor) measuring 30 × 30 × 15 cm and divided in nine squares of equal area was used. Mice were placed inside the box during 5 minutes. During the analysis, the number of squares crossed with all paws, rearing and grooming were counted and used as an indication of locomotor activity [23]. Animals were treated with vehicle (10 mL/kg, i.p.),* C. cordiifolius* essential oil (100 mg/kg, i.p.), or diazepam (1 mg/kg, i.p.) 30 min before the test.

### 2.14. Statistical Analysis

Data was expressed as mean ± SEM and analyzed by ANOVA followed by Dunnett's test using GraphPad Prism 5.0 with significance set at *P* < 0.05.

## 3. Results and Discussion

Ethnobotanical data was collected from one hundred people in the cities of Salgueiro, Terra-Nova, Parnamirim, and Serrita between January and March 2011. Most people interviewed were female (70.5%), between 40 and 59 years old (50.5%), of which 78.8% knew medicinal plants. The most cited plants were “ameixa” (63.5%), “aroeira” (61.6%), and “quebra-faca” (33.0%). After viewing a specimen of* C. cordiifolius*, 98% of the interviewees recognized it as “quebra-faca,” citing many medicinal uses for the treatment of inflammation, pain, wounds, and gastrointestinal disturbs. Medicinal uses, number of citations, and use value of* C. cordiifolius* are listed in Table 1.

Fresh leaves of* C. cordiifolius* were extracted in a Clevenger-type apparatus to obtain the essential oil (0.81%). Determination of the chemical composition of* C. cordiifolius* essential oil (CcEO) performed by GC/MS and NMR analysis revealed the presence of monoterpenes and sesquiterpenes, including oxygenated compounds, similar to other* Croton* species [16]. The composition of the oil, including the retention index and the percentage of each constituent, is presented in Table 2. Analysis by ^1^H and ^13^C NMR and comparison with literature data [24] confirmed the presence of 1,8-cineole (25.09%) and *α*-phellandrene (15.43%) as the major components (see Supplementary Data in Supplementary Material available online at http://dx.doi.org/10.1155/2015/620865). No scientific report concerning the composition or pharmacological properties of CcEO could be found in the literature; however the major compounds 1,8-cineole and *α*-phellandrene have been studied. Several pharmacological activities of 1,8-cineole were described, such as anti-inflammatory and analgesic, antitussive, antitumoral, gastroprotective, and hepatoprotective activities [25]. The antinociceptive activity of *α*-phellandrene was recently reported in murine models of chemical and mechanical nociception [26].

Single dose acute toxicity was assayed through a limit test of 1,000 mg/kg by intraperitoneal route. Five animals were tested. During the 14-day observation period, one animal died. Gross necropsy showed no macroscopic changes in vital organs. As only one animal died, LD_50_ value was considered higher than 1,000 mg/kg. Doses of 50 and 100 mg/kg were chosen for the antinociceptive tests.

**Figure 1:**
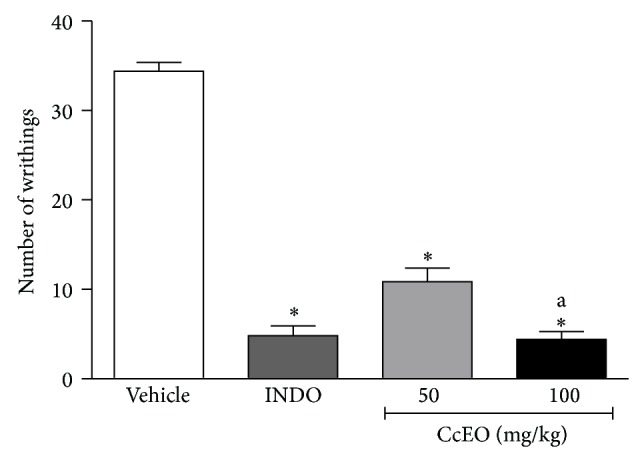
Effect of* C. cordiifolius* essential oil (CcEO; 50 and 100 mg/kg, i.p.) and indomethacin (INDO; 10 mg/kg, i.p.) in the acetic acid induced writhing test. Results are expressed as mean ± SEM (*n* = 8) with significance level set at *P* < 0.05. ^*^When compared to the vehicle group; ^a^when compared to CcEO 50 mg/kg group.

In the acetic acid induced writhing, both doses of CcEO reduced the number of writhing in a dose-dependent manner compared to the control group, as showed in Figure 1. This test is a useful tool to evaluate both central and peripheral analgesic activity of new compounds. Acetic acid induces peritoneal resident cells (macrophages and mast cells) to liberate TNF-*α* and prostaglandin E2, which produce the noxious stimuli. Anti-inflammatory drugs as indomethacin, a cyclooxygenase inhibitor, are effective in reducing pain [27]. Despite the test being sensitive to weak analgesics, it is also sensitive to other pharmacological agents, such as neuroleptics, anticholinergics, and antihistamines [28]. Both CcEO major compounds 1,8-cineole and *α*-phellandrene, when administrated alone, have been shown to be effective in this animal model [25, 26].

**Figure 2:**
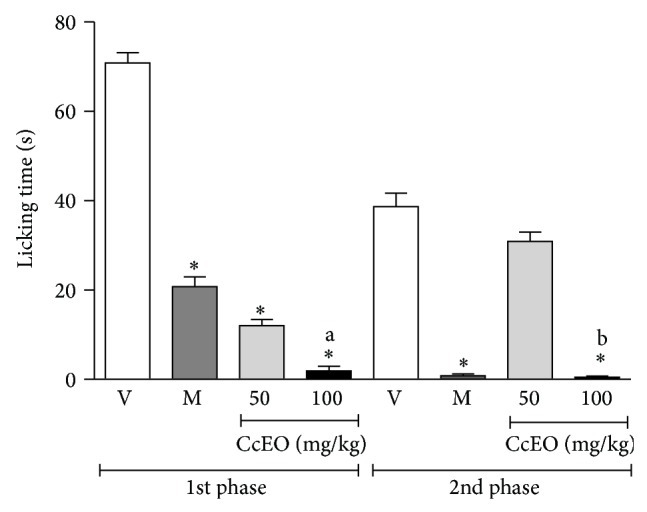
Effect of* C. cordiifolius* essential oil (CcEO; 50 and 100 mg/kg, i.p.) and morphine (M; 7.5 mg/kg, i.p.) in the nociception induced by intraplantar formalin injection. Results are expressed as mean ± SEM (*n* = 8) with significance level set at *P* < 0.05. ^*^When compared to the vehicle (V) group in each phase; ^a^when compared to CcEO 50 mg/kg group in the first phase; ^b^when compared to CcEO 50 mg/kg group in the second phase.

The formalin test can be divided into two phases: the neurogenic phase (or first phase) and the inflammatory phase (or second phase). Neurogenic phase initiates at the moment of formalin injection and lasts for 3–5 min. In this phase, pain is mediated by substance P and bradykinin. The inflammatory phase starts approximately 20 min after formalin injection and lasts for 5–10 min. In that phase amines (histamine and serotonin), prostaglandins, and bradykinin play the major role. Morphine is an effective inhibitor of both phases whereas nonsteroidal anti-inflammatory drugs and steroids are only effective in the inflammatory phase [20]. Here, in the neurogenic phase both doses (50 and 100 mg/kg) of CcEO inhibited the time spent licking the injected paw in a dose-dependent manner. The inhibition in the higher dose was greater than morphine (7.5 mg/kg). In the inflammatory phase, only the dose of 100 mg/kg was effective (Figure 2). Santos et al. [25] and Lima et al. [26] described similar results in formalin test for pure 1,8-cineole and *α*-phellandrene, respectively, emphasizing the role of these compounds in the antinociceptive activity of* C. cordiifolius* essential oil.

**Figure 3:**
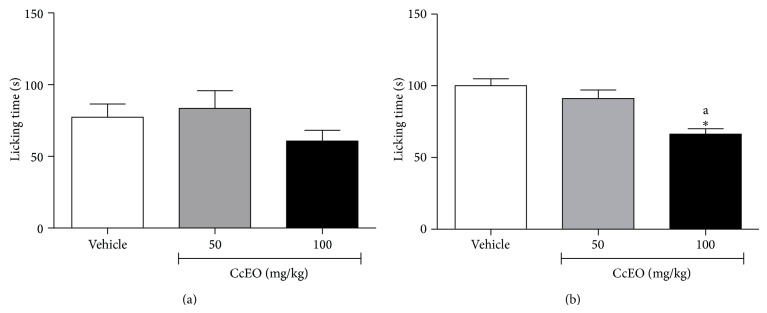
Effect of* C. cordiifolius* essential oil (CcEO; 50 and 100 mg/kg, i.p.) in the nociception induced by intraplantar capsaicin (a) and glutamate (b) injection. Results are expressed as mean ± SEM (*n* = 8) with significance level set at *P* < 0.05. ^*^When compared to the vehicle group; ^a^when compared to CcEO 50 mg/kg group.

In order to elucidate the mechanism of action of the CcEO, the nociception induced by intraplantar injection of capsaicin was tested. Capsaicin is a natural product found in chili peppers (*Capsicum* spp.). Capsaicin is an agonist of TRPV1, an ion channel member of transient receptor potential family, which is enriched in sensory neurons involved in pain perception. TRPV1 can be activated in peripheral neurons by different stimuli, such as heat, acid, vanilloids (e.g., capsaicin and gingerols), and endocannabinoids allowing the transient influx of Ca^2+^ [29, 30]. No effect was observed in both doses of CcEO, as showed in Figure 3(a), suggesting that there was no participation of TRPV1 receptors in the antinociceptive effect of CcEO, despite the antinociceptive effect of pure *α*-phellandrene on this animal model [26].

Another test was performed, this time to evaluate the possible role of glutamatergic system in the antinociceptive properties of CcEO, since this is one of the possible mechanisms of action of *α*-phellandrene [26]. Nociceptive response caused by glutamate involves peripheral, spinal, and supraspinal sites and its action is mediated by NMDA and non-NMDA receptors, as well as by nitric oxide (NO) release [22]. NO increases the synthesis/release of inflammatory mediators such as cytokine, reactive oxygen species (ROS), and arachidonic acid derivatives enhancing the inflammatory reaction and the associated pain [22, 26]. Here, the higher dose of CcEO inhibited the nociception induced by glutamate suggesting the involvement of glutamatergic system in the antinociceptive effect of* C. cordiifolius* essential oil (Figure 3(b)).

**Figure 4:**
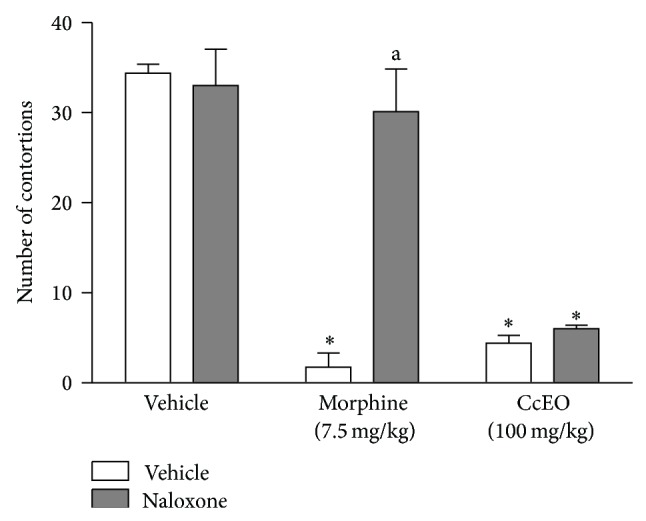
Evaluation of the pretreatment with naloxone (2 mg/kg, i.p.) in the effect of* C. cordiifolius* essential oil (CcEO; 50 and 100 mg/kg, i.p.) and morphine (7.5 mg/kg, i.p.) in the acetic acid induced writhing test. Results are expressed as mean ± SEM (*n* = 8) with significance level set at *P* < 0.05. ^*^When compared to the vehicle group; ^a^when compared to morphine group.

To evaluate the role of endogenous opioids on the antinociceptive effect of CcEO, naloxone, an opioid antagonist was evaluated in another writhing test [22]. The results presented in Figure 4 show that when naloxone (1 mg/kg, i.p.) was given to mice 15 min before CcEO (50 and 100 mg/kg, i.p.) or morphine (7.5 mg/kg, i.p.), the antinociceptive effect of morphine was completely reversed. However, naloxone was not able to modify the antinociceptive effect caused by the CcEO in the writhing test, indicating that the endogenous opioid system is not involved in the antinociceptive activity of CcEO.

The antinociceptive effect of plant extracts and isolated compounds are usually evaluated by behavioral tests. Several drugs as muscle relaxants and sedatives can mask those tests with false positive results [31]. To eliminate this possibility, the open field method was performed. CcEO (100 mg/kg) did not alter the number of squares crossed, rearing and grooming when compared with vehicle. On the other hand, animals treated with diazepam (1 mg/kg, i.p.) reduced all evaluated parameters. These results suggest that CcEO did not have sedative or stimulant effects on central nervous system (CNS) [31].

## 4. Conclusions

In the present work, it was verified that* C. cordiifolius* is well known and used as medicinal plant in the semiarid region of the northeast of Brazil. The main constituents of its essential oil obtained by hydrodistillation were 1,8-cineole and *α*-phellandrene. CcEO showed antinociceptive effect in murine models of chemical induced pain without the participation of endogenous opioid system and TRPV1 channels. No sedative or stimulant effect on CNS was observed in open field test. The effect of* C. cordiifolius* essential oil could be explained, at least in part, by the inhibition of glutamatergic system.

## Supplementary Material

Chemical analysis of the essential oil obtained from the leaves of Croton cordiifolius Baill. (Euphorbiaceae) was performed through gas chromatography-mass spectroscopy (GC/MS) and ^1^H and ^13^C nuclear magnetic resonance (^1^H NMR and ^13^C NMR). In Supplementary material one can found the GC/MS chromatogram and peak report, in addition to ^1^H and ^13^C NMR spectra.

## Figures and Tables

**Table 1:** Medicinal uses, number of citations, and use value of *Croton cordiifolius* Baill. in the central region of state of Pernambuco, Brazil.

Medicinal uses	Number of citations
Inflammation and pain	39
Wounds	23
Intestinal disturbances	19
Liver problems	7
Itching	4
Cold	4
Kidney problems	4
Fever	3
Aphrodisiac	3
Stomach problems	2
Sinusitis	1
Bleeding	1
Diabetes	1

Total citations	111
Use value	1,11

**Table 2:** Chemical composition of the essential oil of *Croton cordiifolius* Baill. leaves.

Volatile constituent^a^	RI^b^	RI^c^	Retention time (min)	Relative area %
Monoterpenes				
*α*-Pinene	939	937	13.88	4.96
*β*-Phellandrene	964	974	16.15	4.68
*α*-Phellandrene	1003	1005	18.01	15.43
*β*-Cymene	1025	1024	19.21	8.02
D-Limonene	1030	1029	19.51	5.22
Oxygenated monoterpenes				
1,8-Cineol	1031	1032	19.73	25.09
*β*-Linalool	1097	1097	23.67	2.34
*α*-Terpineol	1189	1192	29.53	3.41
Sesquiterpenes				
*β*-Caryophyllene	1419	1418	43.41	6.58
*γ*-Elemene	1430	1484	47.49	7.36
Oxygenated sesquiterpenes				
Spathulenol	1578	1518	49.55	6.68
*β*-Caryophyllene oxide	1583	1520	49.69	4.72
Cadinol	1640	1534	50.59	5.51

Total				100.0

^a^Constituents listed in order of elution on DB-5 column.

^b^RI = Kovats retention index according to n-alkanes (C8–C26).

^c^RI = Kovats retention index simulated by equation IK = 16.267RT + 711.48.
