# Supplementary material for: Antinociceptive Effect of the Essential Oil Obtained from the Leaves of Croton cordiifolius Baill. (Euphorbiaceae) in Mice
Source: Evid Based Complement Alternat Med. 2015 Mar 2;2015:620865. doi: 10.1155/2015/620865 (PMC4363708; doi:10.1155/2015/620865)

Supplementary Information

Figure S1. Gas Chromatography-Mass Spectroscopy analysis of *Croton cordiifolius* essential oil. **a.** GC-MS chromatogram, **b.** GC-MS peak report.

a.

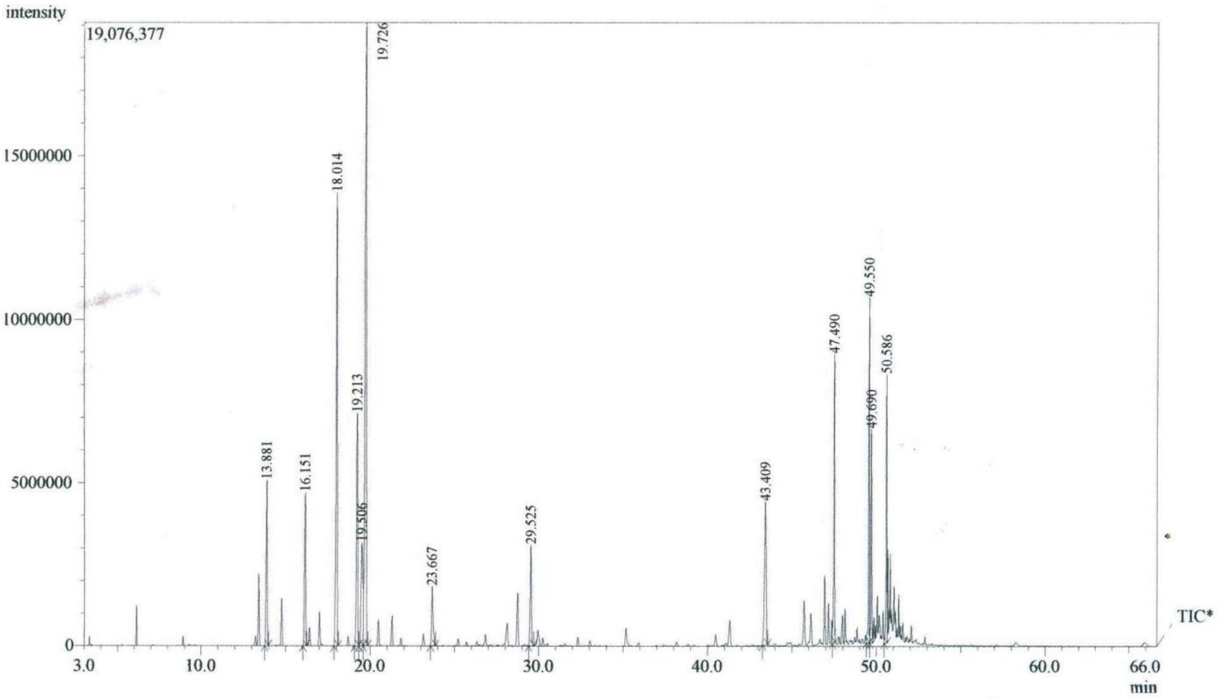

b.

Peak#	R.Time	Area	Height	A/H	Mark	Name	Base m/z	Area%
1	13.881	22835927	5074978	4.50			93.10	4.96
2	16.151	21553164	4696630	4.59			93.10	4.68
3	18.014	71010347	13864954	5.12			93.10	15.43
4	19.213	36886692	7129365	5.17			119.10	8.02
5	19.506	24007099	3166689	7.58	V		68.05	5.22
6	19.726	115469228	19063348	6.06	V		43.05	25.09
7	23.667	10748453	1819513	5.91			71.05	2.34
8	29.525	15690775	3094592	5.07			59.05	3.41
9	43.409	30270014	4386903	6.90			93.10	6.58
10	47.490	33865220	8842316	3.83	V		121.15	7.36
11	49.550	30760973	10571234	2.91	V		43.05	6.68
12	49.690	21700540	6553023	3.31	V		43.05	4.72
13	50.586	25358173	8231568	3.08	V		161.15	5.51
		460156605	96495113					100.00

Figure S2. ^{13}C NMR spectrum of the essential oil of *Croton cordiifolius* (CDCl3, 75MHz).

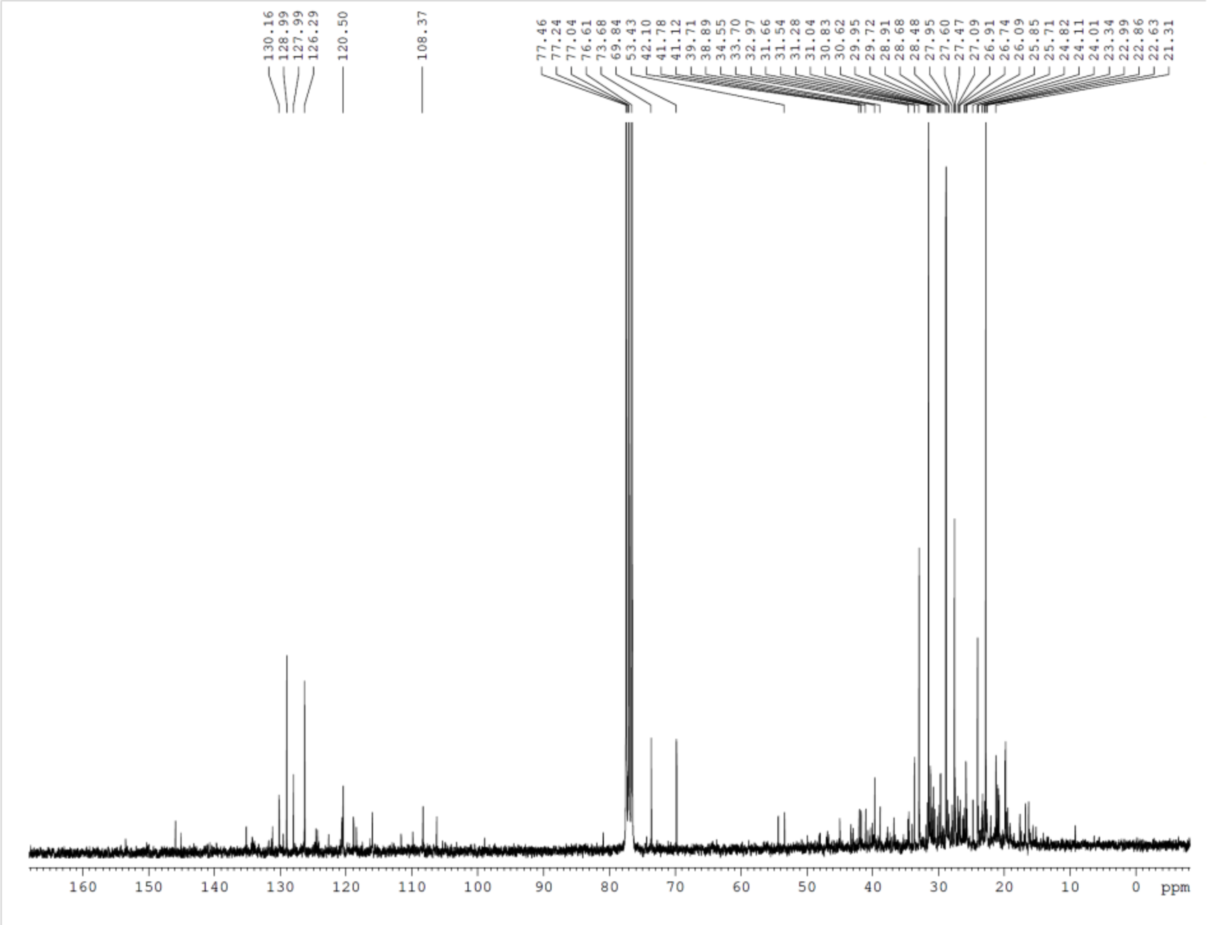

Figure S3. ^{1}H NMR spectrum of the essential oil of *Croton cordiifolius* ($CDCl_3$, 300 MHz).

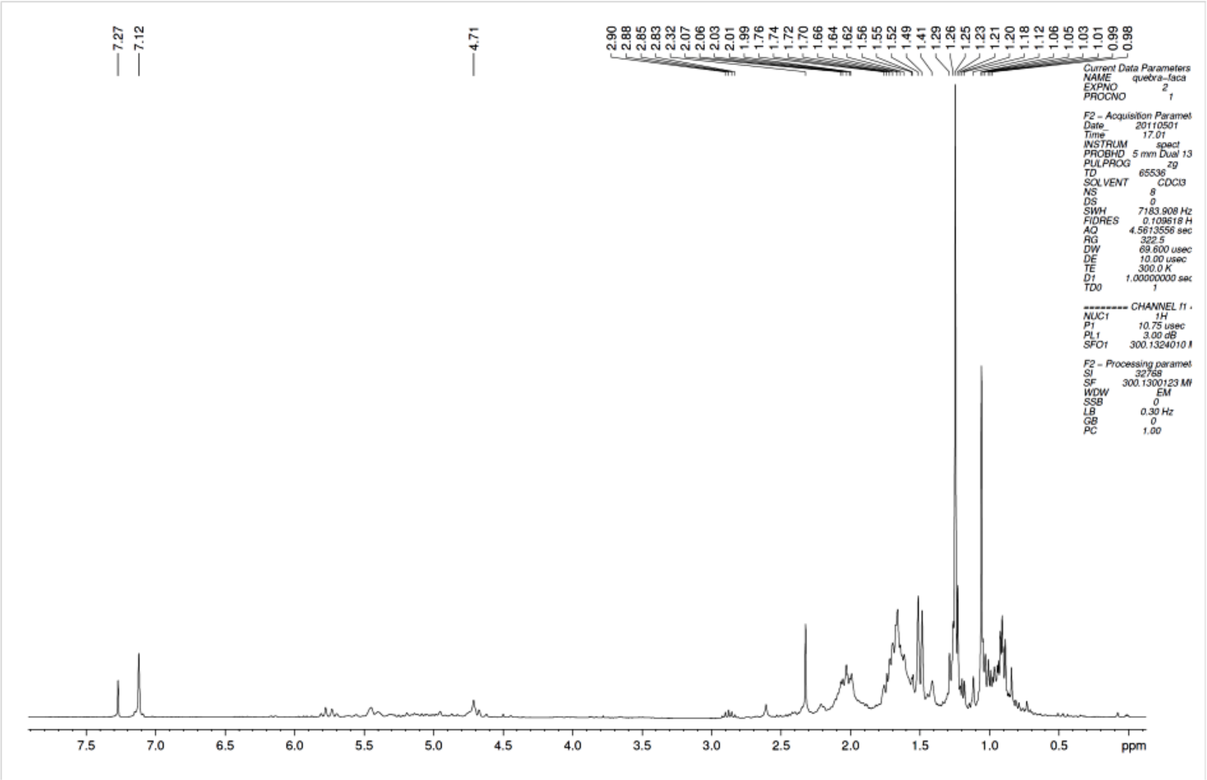

Supplement: Supplementary file 1 — Chemical analysis of the essential oil obtained from the leaves of Croton cordiifolius Baill. (Euphorbiaceae) was performed through gas chromatography-mass spectroscopy (GC/MS) and 1H and 13C nuclear magnetic resonance (1H NMR and 13C NMR). In Supplementary material one can found the GC/MS chromatogram and peak report, in addition to 1H and 13C NMR spectra. [file 620865.f1.pdf]
